# Future Portrait of the Athletic Brain: Mechanistic Understanding of Human Sport Performance *Via* Animal Neurophysiology of Motor Behavior

**DOI:** 10.3389/fnsys.2020.596200

**Published:** 2020-11-17

**Authors:** Eros Quarta, Erez James Cohen, Riccardo Bravi, Diego Minciacchi

**Affiliations:** Physiological Sciences Section, Department of Experimental and Clinical Medicine, University of Florence, Florence, Italy

**Keywords:** information processing, motor control, neural networks, animal models, sport performance

## Abstract

Sport performances are often showcases of skilled motor control. Efforts to understand the neural processes subserving such movements may teach us about general principles of behavior, similarly to how studies on neurological patients have guided early work in cognitive neuroscience. While investigations on non-human animal models offer valuable information on the neural dynamics of skilled motor control that is still difficult to obtain from humans, sport sciences have paid relatively little attention to these mechanisms. Similarly, knowledge emerging from the study of sport performance could inspire innovative experiments in animal neurophysiology, but the latter has been only partially applied. Here, we advocate that fostering interactions between these two seemingly distant fields, i.e., animal neurophysiology and sport sciences, may lead to mutual benefits. For instance, recording and manipulating the activity from neurons of behaving animals offer a unique viewpoint on the computations for motor control, with potentially untapped relevance for motor skills development in athletes. To stimulate such transdisciplinary dialog, in the present article, we also discuss steps for the reverse translation of sport sciences findings to animal models and the evaluation of comparability between animal models of a given sport and athletes. In the final section of the article, we envision that some approaches developed for animal neurophysiology could translate to sport sciences anytime soon (e.g., advanced tracking methods) or in the future (e.g., novel brain stimulation techniques) and could be used to monitor and manipulate motor skills, with implications for human performance extending well beyond sport.

## Introduction

Numerous sport performances appear esthetically appealing and deceptively simple. At the heart of such performances are complex dynamics involving body mechanics and neural control. Here we argue for a stronger interaction between sport neuroscience and non-human (henceforth simply animal or basic) neurophysiology, to provide mutual benefits for both disciplines, i.e., behavioral outcome in sport and cellular mechanisms in animal studies, toward a deeper understanding of the nature of motor performance.

Indeed, many sports gestures can be regarded as prominent showcases of skilled motor control and thus, considering the central nervous system (CNS) as a machine producing adaptable movements, are of great relevance for various disciplines including cognitive neuroscience (Shadmehr and Krakauer, 2008; Graziano, 2009; Yarrow et al., 2009). Unsurprisingly, investigations on the neural bases of sport performance raised interest also in human physiology and biomedicine. Classic physiological research focused, among others processes, on fatigue, with the long-dominating view considering it a muscular limit. This view is now partially disrupted in favor of evidences indicating that, at the bottom of muscular fatigue, there is also an exhaustion of the nervous system (Noakes, 2012). In biomedicine, sport was assessed mostly as either a health-promoting or harming intervention. In the first case, sport serves to model increased level of physical activity, with a typical intervention goal being prevention of non-neural pathologies associated with sedentary lifestyle, while a more recent focus has been to use sport as a way of promoting “brain health” (Boecker et al., 2012; Malm et al., 2019; Logan et al., 2020). In the second case, sport entails increased risks for traumatic CNS injuries (especially sports like Boxing or American Football), and it is possible to predict the magnitude of the behavioral impairments based on the intensity of the head impacts (Gavett et al., 2011; Castellani and Perry, 2017; Mckee et al., 2018; Leeds et al., 2019). Also, epidemiological data support the hypothesis that some athletes, like football players, have an increased risk of developing certain neurodegenerative diseases, including possibly amyotrophic lateral sclerosis (Blecher et al., 2019).

A more recent research line aims to investigate the neural bases of motor performance, and a first milestone has been to uncover behavioral and neural differences between naive and expert athletes, contributing to the establishment of sport neuroscience (Yarrow et al., 2009; Carlstedt, 2018). This new field leverages mainly upon concepts and methodologies of sport psychology and cognitive neuroscience (Milne, 2007; Gee, 2010; Zhou and Zhou, 2019), and the integration of methods and/or concepts emerging from neurophysiological studies will likely provide a groundbreaking stimulus toward a mechanistic understanding of the neural bases of human performance.

## On the Definitions of Sport

While in some contexts, physical activity and exercise (Caspersen et al., 1985) are terms used interchangeably with the term sport, for the latter we embrace the following definition: “an activity involving physical exertion and skill, especially one regulated by set rules or customs in which an individual or team competes against another or others” (Oxford English Dictionary, 2020). Also, the effects of physical activity and exercise at the neural level are already well established in human and animal studies; sport has lagged behind. In addition, sport is an umbrella term encompassing disparate disciplines associated with quite heterogeneous sets of cognitive and motor abilities. Broadly, two sport categories can be distinguished, namely, closed skill sports (CSS) and open skills sports (OSS), whereby the first category includes those sports that are often based on the alternate and rhythmic repetition of limb movements, where the context is relatively constant and predictable (e.g., swimming) (Wang et al., 2013a). At variance, in OSS the athlete’s performance is embedded in an environment that is dynamic, difficult to predict, and externally paced (e.g., tennis) (Wang et al., 2013a; Bove et al., 2017). Hence, while a CSS also involves central networks associated to, e.g., energy management, most CSSs are mainly based on variation of locomotion parameters and thus could be regarded as “less cognitive” and more associated with low-level motor control and circuits (i.e., spinal) (Wang et al., 2013a). In OSS, on top of fine-tuning of locomotion parameters, other aspects, such as skilled object manipulation, action observation and anticipation, and a coral, tactical strategy, are necessary to succeed. It may thus seem intuitive to consider animal modeling of, e.g., running to be less complex than, say, tennis and that differential insights on the cognitive bases of sport performance may be extracted. Here, we exclude sports involving the active involvement of animals (such as horse racing).

In addition to canonical sports, competitive video gaming (Winkie, 2019) is emerging as a new sport discipline termed electronic sport (eSport). Movements of eSport athletes are usually constrained to keystrokes, gamepads, joystick, and mouse movements, which facilitates, compared to many real world sports, hypothesis testing and task manipulations typical of laboratory-based experimentation. Critically, virtual reality studies are routinely performed in both humans and animals and could be readily adapted to model eSport, which could be harvested toward a mechanistic understanding of motor performance (Sousa et al., 2020). Whether eSport can be considered as a “true” sport is still debated (Parry, 2019); however, it is worth mentioning that eSport-related activities are on the verge of being incorporated in future Olympics (Grohmann, 2017). Independently of such organizations’ regulations, eSports could be an important research tool for assessing cognitive processes underlying some aspects of sport performance, similar to what has been done using, e.g., flight simulations for military training/testing. Future research will establish the extent to which spatial scaling of the motor effector used in virtual versus physical environments could influence the performance and/or whether scale-invariant parameters [possibly obeying the fractal ordering principle, (Turvey and Fonseca, 2009; Michalski et al., 2019)] emerge. Some initial indications come from mice studies, where virtual reality tasks are associated with partially altered hippocampal dynamics compared with a real-world task (Aghajan et al., 2015), implicating that similar variation in neural computation may occur in humans. The fidelity with which virtual reality settings, including eSports, can emulate aspects of physical sports remains an active field of research. In the case of CSSs, for instance, cycling, a partial convergence between these two worlds (electronic/virtual and physical/real), has already taken place. For instance, commercial systems allow integrating the use of a stationary bike with the rear wheel placed onto a motorized roller, whose bidirectional communication with a computer permits to adjust the resistance and the virtual landscape (Lazzari et al., 2020). Achieving such convergence in the case of OSSs is more challenging; however, encouraging results have been obtained by showing that free throw in basketball can be improved when subjects are trained in a virtual reality simulator (Covaci et al., 2012). We address readers to some recent extensive reviews of this field (Campbell et al., 2018; Akbaş et al., 2019) for a more dedicated appraisal on the subject of eSports virtual reality applied to sports. In the future, it will be interesting to assess neural parameters using an approach similar to the one used in the rodent study mentioned above, that is, examining subjects in both environments, to assess for potential neural similarities/differences in the physical versus virtual environment.

Based on the above premises on sports-specific characteristics, a body of work has tested the hypothesis that behavioral and neural processes display variations not only between athletes and non-athletes but also between athletes from different sports [e.g., CSS vs. OSS (Kizildag and Tiryaki, 2012; Wang et al., 2013b)], or between naive subjects, professional athletes, and elite athletes, the latter acting as statistical outliers in terms of sport performance (Aitken, 2004; Hardy et al., 2017). In the following section, we discuss some recent work encompassing these levels of investigation and relate some of these findings to laboratory-based studies of human motor performance.

## Ready, Set, Go! on Cognitive and Neural Features of Athletes

By definition, in sport contexts, the subjects’ performances are pushed to the limit and as such may teach us critical principles of human expert behavior (Walsh, 2014). If these performances are considered as complex (individual and/or interpersonal) acts, then their study places them at the core of emerging concepts in neural sciences, including embodied cognition theories, which state that “cognition should be described in terms of agent–environment dynamics rather than computation and representation” (Chemero, 2020). While shortcomings of such approach are self-evident, re-evidencing the role of the body as well as the environment for a deeper understanding of the brain may have its merits. From an anatomophysiological point of view, investigating on athletes, like musicians in the artistic setting (Münte et al., 2002), is informative for learning about neuroplasticity and maladaptive plasticity resulting from aberrant training of a specific motor action [mostly through cross-sectional studies, and some longitudinal studies (Ioannou et al., 2018; Bravi et al., 2019)].

Because sport neuroscience is a relatively new field, a characterization of cognitive performances and their neural bases in athletes can be considered still in its infancy. Nonetheless, what is required to be successful at the highest level in sport is intuitively a multifaceted set of cognitive abilities. We readdress on this regard interested readers to pertinent reviews (Yarrow et al., 2009; Nakata et al., 2010) or books (Boecker et al., 2012; Carlstedt, 2018) and mention here only a few striking instances linked to the above described categories (athlete vs. non-athlete, CSS’s athlete vs. OSS’s athlete, normal vs. elite athlete).

Among the behavioral parameters shown to be modified in athletes, inhibitory control (Benedetti et al., 2020), i.e., the suppression of inappropriate behavioral responses, is improved in elite athletes (Brevers et al., 2018), and there is a robust difference among players of OSS vs. CSS, with the former outclassing the latter (Wang et al., 2013a). Skilled athletes can predict the outcome of actions performed by others, based on the kinematic information inherent in others’ actions, earlier and more accurately than less-skilled athletes (Aglioti et al., 2008; Unenaka et al., 2018). Also, proactive control was also evidenced to be modulated by motor skill experiences, with OSS athletes showing higher levels of efficiency than CSS athletes (Yu et al., 2019).

Not surprisingly, some of the behavioral traits of motor know-how are complemented by anatomical evidence. For example, corpus callosum is thicker in expert performers (Gooijers and Swinnen, 2014; Meier et al., 2016). Concerning neurophysiological data, signals obtained from humans with, e.g., electroencephalography (EEG) or functional magnetic resonance imaging (fMRI), show activity related to movement and motor expertise, such as reduced brain activation in experts (neural efficiency) (Guo et al., 2017; Del Percio et al., 2019) and different threshold to elicit corticospinal facilitation (Fomin et al., 2010; Fomin and Selyaev, 2011; Wang et al., 2014; Wright et al., 2018).

While EEG has lower spatial resolution with respect to methods like fMRI and functional near-infrared spectroscopy, it provides superior temporal resolution and is thus more suitable for investigating neocortical activation patterns associated with fast (i.e., in the millisecond range) movements typical of sports. While other methods such as magnetoencephalography (Mäkelä, 2014) and event-related optical signal (Gratton and Fabiani, 1998) have a comparable temporal resolution, EEG-based investigations have been far more frequently applied to sport performance. In addition, the rise of portable EEG devices further offers an invaluable opportunity to study sport gestures outside laboratory settings (Park et al., 2015; Wang et al., 2019). We thus restrict our focus on some relevant EEG studies. The most common approaches are based on comparisons such as preperformance vs. movement execution, good vs. bad performance, expert vs. novice, competitive vs. non-competitive athletes, disabled vs. non-disabled athletes, baseline vs. learning, and practice vs. competition. Two main categories of movement responses are usually investigated in EEG studies of motor performance, namely, movement-related potentials, including Bereitschaftspotential (readiness potential) and motor potential, and action-monitoring potentials, such as error-related negativity (Carlstedt, 2018). A well-known frequency-domain manifestation of movement includes the Mu rhythm, a decrease of alpha band and beta band power occurring in the sensorimotor regions of the neocortex during movement preparation (Jenson et al., 2020), and OSS athletes (karate and fencing) compared to control subjects display reduced alpha band activity even during simple upright standing (Del Percio et al., 2009). Reduced activity in the alpha band has been reported in CSS athletes (cyclists) as well, which would suggest that enhanced neural efficiency does not depend on the type of OSS or CSS sport category practiced (Ludyga et al., 2015). In contrast, a bilateral increase in parietal areas has been reported in football players during action observation (Del Percio et al., 2019). Within the time domain of EEG signals, motor expertise (fencers) has been linked to altered event-related potentials and faster stimulus discrimination during go–no-go tasks (Di Russo et al., 2006). Faster reaction time is associated with shorter readiness potential in athletes (baseball players) during go trials, while in no-go trials, they display an augmented P300 amplitude in the frontal regions, implying that improved stimulus responses depend on faster response selection and more robust inhibition (Nakamoto and Mori, 2008). In a similar fashion, other OSS experts (table tennis player) exhibit superior response inhibition compared to non-athletes (Yu et al., 2019).

Growing evidence suggests that baseline cognitive ability could be used to predict future achievements in sports, and studies have shown that both core and higher-level executive functions predict the success of athletes (Vestberg et al., 2012, 2017; Mangine et al., 2014). The level of expertise is also expressed by a sort of “immunity” against distractive stimuli in elite athletes: novice athletes are affected strongly by distracting tasks, whereas experts are shielded against this distraction, indicating highly automatic performance (French et al., 1995; Gray, 2004; Yarrow et al., 2009).

Interrogating neural networks in humans implies limited access to cellular, spiking data *in vivo*, excluding extracellular recordings during neurosurgeries, and even then, the role played by different classes of neurons can only be indirectly inferred. Besides monitoring the activity of brain networks, to determine a causal role of a given neural pattern for performance, manipulation techniques, such as transcranial magnetic stimulation and transcranial direct current stimulation (tDCS), are advancing rapidly from clinical settings to sport (Goodall et al., 2014; Edwards et al., 2017; Gazerani, 2017). Interestingly, such methods provide an opportunity to improve sport performance. Endurance performance is increased in recreationally active participants after anodal, but neither cathodal nor sham, bilateral stimulation of motor cortices, and this effect is associated with increased corticospinal excitability of the knee extensor muscles and reduced perception of effort (Angius et al., 2018). Aside from ethical aspects associated with the possibility of stimulation techniques to become part of enhancement tools known as neurodoping (Davis, 2013; Kamali et al., 2019), and the fact that the long-term effects of brain stimulation are unknown, the possibility of increasing performance by refining brain stimulations methods is an exciting, although controversial, area of research. Technically, major limitations of current methods are the coarse spatial resolution and that the stimulation is not cell type–specific. Hence, efforts to improve our understanding of the neurophysiology could help develop more efficient approaches in sport settings.

For both monitoring and stimulating neural activity, animal models offer the opportunities to dissect, within a reverse engineering approach, brain circuits to determine the causal role of specific patterns and develop novel neurotechnologies well beyond the state of the art available in humans. In the following section, we discuss examples from animal research that could contribute, from a conceptual and/or a methodological stance, to gain a more fine-graded understanding of the neuronal basis of athletic performance.

## Observing and Hacking the Animal Brain During Motor Behavior

Songbirds (Clayton, 2019), rodents (Makino et al., 2017; Hwang et al., 2019; Quarta et al., 2020, *preprint article*; Sauerbrei et al., 2020), and non-human primates studies (Churchland et al., 2012; Bruni et al., 2017; Filippini et al., 2018; Battaglia-Mayer and Caminiti, 2019) provide valuable insights into the neurophysiology of motor skills; however, sport science has paid relatively little attention to these mechanisms of expert behavior. For example, neural recordings from finches have been classically used to investigate the dynamics of motor learning by imitation (Roberts et al., 2012), which is a learning approach at the core of sport performances in developing athletes (Unenaka et al., 2018). In this regard, a potentially important role is played by the well-known mirror neurons, discovered in the premotor and posterior parietal cortex of NHPs (Ferrari and Rizzolatti, 2014). Importantly, mice improved their acquisition of a simple operant conditioning task by observational learning, with medial prefrontal cortex and the nucleus accumbens significantly involved in the acquisition and proper task performance (Jurado-Parras et al., 2012). Driven by results arising from animal studies, experiments on the role played by motor imagery training for sport performance have gained momentum (Lewthwaite and Wulf, 2010).

Research lines on the role played by physical activity for the homeostasis of neural circuits and behavior are now well established in rodents. As a notable instance, specific physical exercise protocols in rodents, for example, running, have been repetitively associated with enhanced levels of neuroplasticity and improved behavioral learning (van Praag et al., 1999; Kobilo et al., 2011; Li and Spitzer, 2020).

Rodents have recently gained momentum to investigate certain aspects of motor performance, and it is now established that cellular actors including neurotrophins such as brain-derived neurotrophic factor (BDNF) mediate motor skill learning (Arango-Lievano et al., 2019). For example, it was demonstrated that BDNF signaling is necessary for the behavioral effects of tDCS to occur (Fritsch et al., 2010). Importantly, in the same work, the authors extended their findings to humans, demonstrating a limited effect of tDCS stimulation in subjects with a polymorphism known to reduce [18–30% (Egan et al., 2003; Chen et al., 2006)] the secretion of BDNF, implying that the effects are likely mediated by this type of cellular signaling in humans as well (Fritsch et al., 2010). In any case, the relationship between motor behavior and BDNF signaling has been under intense scrutiny in both rodents (Boger et al., 2011; Besusso et al., 2013) and humans (Grégoire et al., 2019).

Selected types of sensorimotor transformations, such as locomotion, are beginning to be understood at the cellular level in rodents (Ferezou et al., 2007; Papale and Hooks, 2018). The investigation of the neural dynamics subserving more complex movements such as reaching and grasping, which form the motor building blocks for many sports gestures, is classically studied in NHPs and more recently adapted for rodents (Guo et al., 2015). In rodents, which allow precise neurophysiological dissection, thanks to the availability of powerful genetic engineering and optical methods, a cortical characterization has been recently made available (Guo et al., 2015; Wang et al., 2017; Quarta et al., 2020, *preprint article*; Sauerbrei et al., 2020).

The relevance of neocortical circuits for manipulative behaviors in rodents has been classically established *via* lesion or pharmacological approaches. For instance, local anesthetics injected in the forelimb area of mice were shown to alter movement parameters (Estebanez et al., 2017; Galiñanes et al., 2018). Also, recent evidence shows a direct involvement of the facial area of the rabbit motor cortex in the acquisition and performance of conditioned eyeblinks (Ammann et al., 2016).

Technological development, most notably *in vivo* optogenetics, opened the opportunity to perform cell type–specific, reversible, and temporally precise (in the millisecond range) excitation or inhibition of neurons in behaving animals, at times with a spatial resolution allowing to dissect the specific role of a given cellular (sub) population (Fenno et al., 2011; Chen et al., 2018).

Using such a method for the study of motor behavior, it was, for instance, discovered that inhibitory neurons in the contralateral sensorimotor cortex command specific phases of reaching and grasping in the mouse (Guo et al., 2015), that cerebellar anterior interposed nucleus exerts control over the speed of reaching movement (Becker and Person, 2019), and that perturbing the thalamocortical communication impairs forelimb movement kinematics in a frequency-dependent manner (Sauerbrei et al., 2020).

Nevertheless, a neuroanatomical limit of comparison of motor circuits between rodents and primates is the corticomotoneuronal pathway, which is thought to serve fine movements in NHPs and humans (Fetz et al., 1989). While tract-tracing experiments could evidence a direct corticospinal connection in rodents with concurrent physiology consistent with corticospinal cells (Sheets et al., 2011; Oswald et al., 2013), current evidence indicates no functional contacts between corticospinal axons and motoneurons in adult rodents (Alstermark et al., 2004). In their seminal work, Alstermark and colleagues also demonstrated that in rodents this pathway is polysynaptic, with additional cell types located in the reticular formation, as well as due to segmental interneurons in spinal cord (Alstermark et al., 2004). A direct corticomotoneuronal pathway could be recently maintained in adult mice *via* genetic engineering, and when this tract is present, their manual dexterity is improved (Gu et al., 2017).

On the other side, a major effort has been made to translate advanced tools for neural circuit interrogation from phylogenetically lower species such as rodents to NHPs (Galvan et al., 2017; O’Shea et al., 2017). Remarkable results have been achieved; for instance, it was demonstrated that dendritic activity recorded optically from the motor cortex of monkeys transfected to express a fluorescent activity reporter in excitatory neurons could reliably be employed to predict the direction of the arm movement [Trautmann et al., 2019, *preprint article*]. Manipulating cerebellar neurons *via* optogenetics could drive saccade movements (El-Shamayleh et al., 2017), while performing similar recordings and stimulation in the motor cortex of marmoset monkeys has been employed to investigate the neural dynamics of arm movements (Ebina et al., 2018, 2019).

In summary, animal models, in particular, rodents and NHPs, offer the opportunity to investigate mechanistic aspects of behavioral expertise (Mayse et al., 2014).

## Of Mice and Men: Examples Toward Animal Models of Sport

While we acknowledge that not all aspects of sport performance will benefit from inputs from animal neurophysiology of motor behavior, we discuss below successful examples that may spark discussion across disciplines.

### Optimal Arousal for Optimal Performance

A remarkable example of successful translation of concepts from animal studies to human performance is represented by the pioneering work by Yerkes and Dodson on the optimal level of arousal needed to achieve the highest performance. In their study, rats were requested to solve an easy or a difficult task and were given a motivational varying cue of different intensity based on errors they made during training (Yerkes and Dodson, 1908). Upon increasing stimulus intensity, the performance of the rats increased monotonically for easy or well-learned task, while for a task considered difficult the performance decreased abruptly when the stimulus intensity exceeded a certain threshold. The experimental data on the latter fitted well a parabolic function and led to the formulation of the Yerkes–Dodson law, stating an inverted-u relationship between arousal and behavioral performance. Since then, similar conclusions were achieved in humans, including in sport settings, and this relationship is related to the well-known phenomena of clutching and of choking under pressure (Kamata et al., 2002; Yu, 2015).

### The Playing Rats

Sport, even in its most competitive settings, has a hedonic motivation. It has been long thought that the latter is an almost exclusive trait of humans; however, recent evidence suggests that even rodents engage in a task “just for fun of it;” Brecht and collaborators were able to demonstrate that rats can play hide-and-seek with a human (Reinhold et al., 2019). Rats quickly learned the game and learned to alternate between hiding versus seeking roles, with specific neural activity patterns emerging in the prefrontal cortex. Clearly, these findings have important implication for the goal of modeling sport (with hide-and-seek being an unusual, yet a candidate Olympic sport) performance in animals, including potential next steps such as optogenetic experiments to alter the activity in the prefrontal cortex to determine the necessity of specific patterns for behavioral performance.

### Motor Skills of an Olympic Mouse: Too Far Reaching?

**FIGURE 1:**
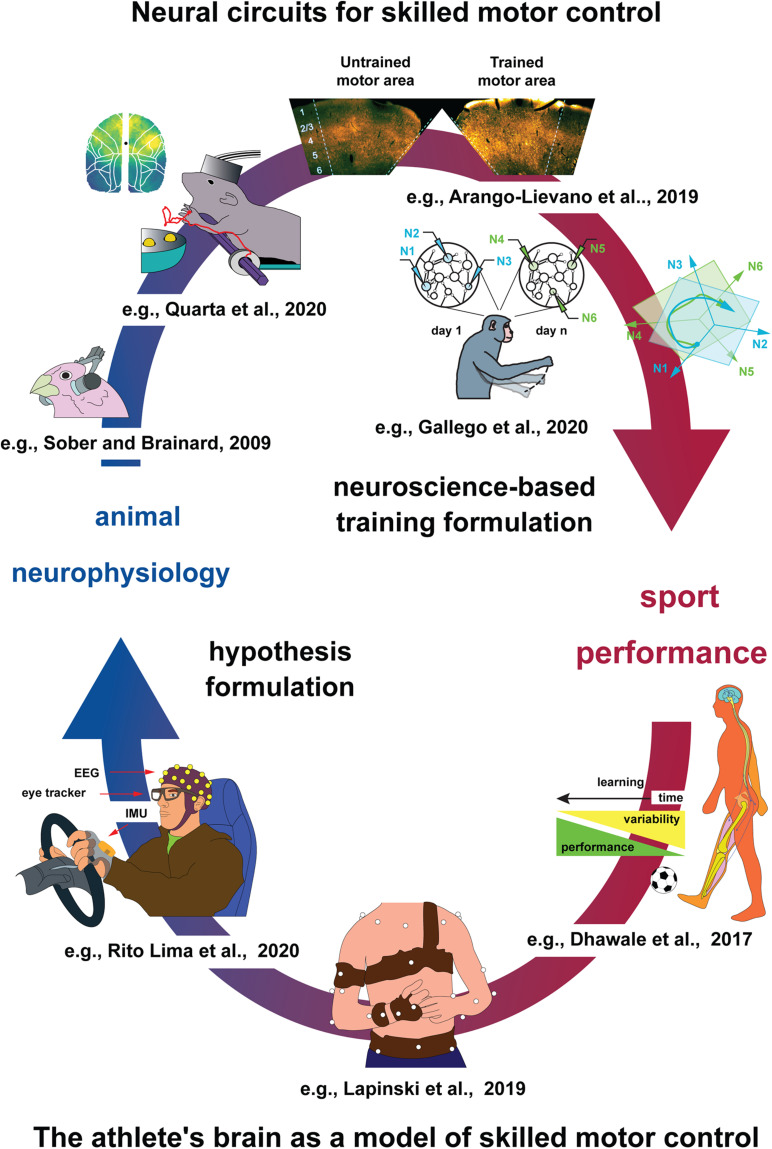
Proposed cycle of information sharing between animal neurophysiology and sport science. *Top: Animal models of skilled motor control*. From left to right. Considering skilled behavior largely depends on commands issued by the nervous system, efforts to shine light on such mechanisms may lead us to a better understanding of sport performance. Animal models allow dissecting such mechanisms in much higher detail, compared to humans. For example, selected neuronal mechanisms for motor learning by imitation can be investigated already in phylogenetic older species such as birds [e.g., finches (Sober and Brainard, 2009; Garst-Orozco et al., 2014; Sober et al., 2018)]. The neural control of limb movements in mammals is increasingly investigated in laboratory rodents, especially rats and mice, which employ powerful optical and genetic tools for cell type–specific analysis of neural dynamics, while permitting to carry out increasingly complex motor tasks (Guo et al., 2015; Ash et al., 2017, *preprint article*; Arango-Lievano et al., 2019; Quarta et al., 2020, *preprint article*). The closest experimental organisms to humans, non-human primates (NHPs), have the highest manual dexterity and still allow single-cell-level interrogation of neural activity during skilled motor control (Gallego et al., 2020). *Bottom: The athlete’s brain as a model of skilled motor control.* From right to left. By integrating both concepts and technologies originally developed in animals, advanced analysis of human sport performance metrics allows multivariate data analysis and hypothesis formulation to be tested in athletes, also in laboratory settings involving behavioral tasks mimicking sport gesture (Dhawale et al., 2017), using marker-based and, increasingly, markerless approaches (Mathis et al., 2018; Lapinski et al., 2019). Movement data, acquired also *via* inertial measurement units and eye trackers, are complemented by, e.g., EEG, which permit coarse-resolution level analysis of neural networks involved in skilled motor control (Rito Lima et al., 2020), serving as a potential starting point for animal studies.

It seems intuitive to reject the hypothesis that a trained animal may be informative about how motor skills emerge in sportspeople, possibly because “being the best of the best as an athlete encompasses more than having a very high level of motor skill after a lot of training” (Krakauer, 2017). However, this limit may be due not to biology *per se* (Grandin and Whiting, 2018) but is rather associated with the common research methodology concerning motor control in animals, which tends to focus on population average (mean motor performance) rather than on the upper statistical outliers (elite motor performance). As a potentially relevant point, genetic tools and selective breeding (e.g., for longer legs) available in animal neurophysiology allow to perform hypothesis testing difficult to perform in humans (e.g., the importance of a genetic background), informing us about the relative importance of specific traits for motor performance. This intended mutual information exchange is drawn as a self-feeding cycle (Figure 1).

## Discussion: The Roadmap for a Transdisciplinary Dialog

To stimulate a transdisciplinary dialog, a back-translation of sport sciences findings to animal models and the evaluation of comparability between animal models of a given sport and athletes will require several intermediate steps. Behaviorally, non-invasive tools developed in animal research will most likely be employed in sport settings anytime soon, in particular methods for markerless tracking based on machine learning approaches, which evidence that detailed information on motor behavior can be extracted from animal and humans with the same approach (Mathis et al., 2018).

**FIGURE 2:**
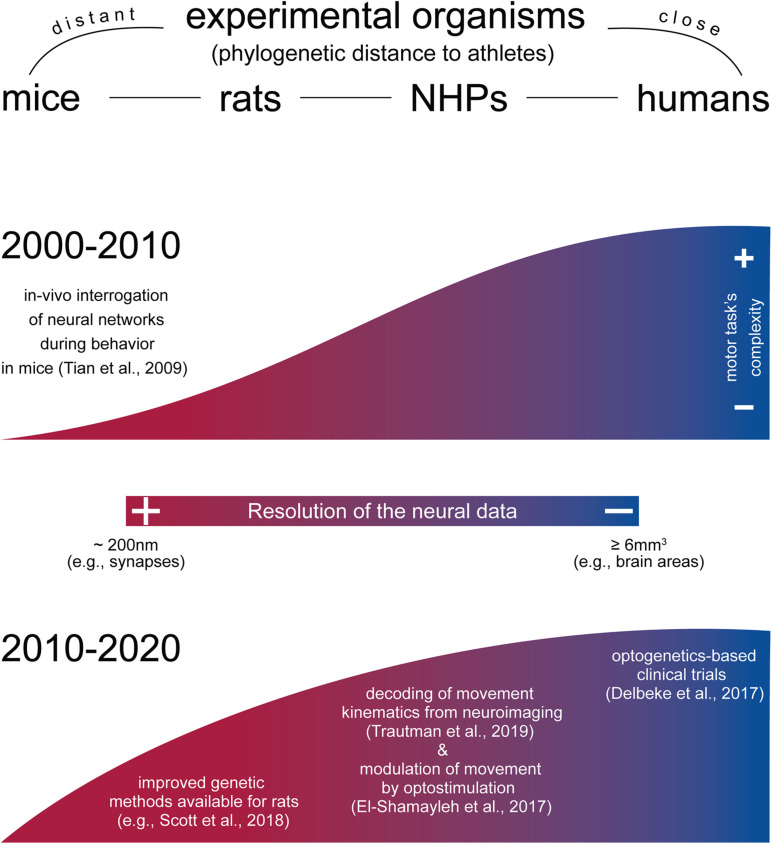
Approximate resolution of neural and behavioral data across experimental organisms. While species-specific morphological and phenotypical characteristics largely separated the motor behaviors one can model in animals, mammals such as mice and rats share remarkable similar motor traits compared to humans (e.g., reach and grasp movements). In this regard, there have been efforts to model progressively more complex movement in animal models during the last decade (graphically represented here as the height of the graph). Concomitantly, a top-down approach has permitted to obtain increasingly better spatiotemporal resolution on the neural dynamics during motor behaviors in NHPs and humans (graphically represented here as the gradient of the graph). Organisms are ranked by phylogenetic distance to athletes, from left to right. Height of the upper and lower graphs approximate visually the motor task complexity obtained across organisms, with the human as gold standard to which the animal models compare. The color bar at the center (resolution of the neural data) refers to the ability of the methods most commonly used in each organism to discriminate increasingly fine structures (from neural areas to single neurons) and/or events (e.g., spike trains). Upper graph, period 2000–2010. In this period, novel physiological methods became widely used to investigate neural activity also at single-cell level in behaving mice (Tian et al., 2009; Yang and Yuste, 2017). Lower graph, period 2010–2020. Methods for neural interrogation became gradually adapted for rats (Igarashi et al., 2018; Scott et al., 2018) and NHPs (El-Shamayleh et al., 2017; Galvan et al., 2017; Kondo et al., 2018; Trautmann et al., 2019, *preprint article*), with first clinical trials in humans (Delbeke et al., 2017). Concomitantly, the complexity of the motor task for rodents became higher (Guo et al., 2015; Quarta et al., 2020, *preprint article*; Sauerbrei et al., 2020).

Encouragingly, in recent years, there has been a successful effort to translate neurophysiological techniques that allow cell type–specific interrogation from rodents to NHPs. Thus, at least in theory, it is procedurally feasible to extend this range of techniques in humans as well. As a notable instance, in the last 10 years, optogenetics has moved from rodent to NHPs for basic neurophysiology studies and has entered preclinical trials in human patients (Simunovic et al., 2019; Figure 2).

An intriguing intermediary step could be the opportunity of investigating the dynamics of human neurons *in vivo* by transplanting induced pluripotent stem cell–derived neurons into the mouse brain (Real et al., 2018). While an investigation on expert motor behavior is yet prospective, this type of xenotransplants could inform us about the mechanisms underlying the neural bases of (sport) performance in a subject-specific manner.

Overall, investigating athletes and trained animals with a logic comparable to early cognitive neuroscience studies on neurologically impaired individuals (Agis and Hillis, 2017) will not only teach us about general principles of behavior but could rather provide a bedrock for novel and more efficient training and rehabilitation methods (Reiman and Lorenz, 2011). This would be conceptually similar to a main use of animal models in biomedical research, that is, to inform us about the mechanisms through which neurodegenerative disorders affect neural circuits and behavior and to test potential new treatments and/or neuroprotective agents, such as neurotrophic factors, physical exercise, and, increasingly, motor expertise (Cai et al., 2014; Quarta et al., 2015, 2018; Nie and Yang, 2017; Dawson et al., 2018; Ng et al., 2019; Tsai et al., 2019).

Thus, an exciting dawn of opportunities lies ahead, which will allow to control, and possibly improve, movements in human subjects extending the limit of human performance (Triviño, 2014).

## Data Availability Statement

The original contributions presented in the study are included in the article/supplementary material, further inquiries can be directed to the corresponding author.

## Author Contributions

EQ and DM: concept development, figure preparation, manuscript preparation, and manuscript proofreading. EC and RB: concept development, figure preparation, and manuscript proofreading. All authors contributed to the article and approved the submitted version.

## Conflict of Interest

The authors declare that the research was conducted in the absence of any commercial or financial relationships that could be construed as a potential conflict of interest.
